# Hijacking the BAF complex: the mechanistic interplay of ARID1A and EWS::FLI1 in Ewing sarcoma

**DOI:** 10.1002/1878-0261.13742

**Published:** 2024-09-29

**Authors:** Erich J. Sohn, David S. Libich

**Affiliations:** ^1^ Greehey Children's Cancer Research Institute and Department of Biochemistry and Structural Biology The University of Texas Health Science Center at San Antonio San Antonio TX USA

**Keywords:** BAF complex, biomolecular condensate, Ewing sarcoma, EWS::FLI1, prion‐like domain

## Abstract

Ewing sarcoma, an aggressive pediatric cancer, is driven by the EWS::FLI1 fusion protein, which disrupts gene expression by hijacking the BAF chromatin remodeling complex. Central to this mechanism is the formation of biomolecular condensates, mediated by the prion‐like domains (PrLDs) of EWS and ARID1A, a core BAF subunit. ARID1A serves as a critical interface between EWS::FLI1 and the BAF complex, with its condensate‐forming ability essential for the aberrant gene expression that drives tumor growth. The loss of condensate‐competent ARID1A significantly impairs tumor progression, identifying it as a potential therapeutic target. However, targeting condensate formation is challenging due to the transient nature of the interactions involved, complicating the development of effective inhibitors. This work underscores the importance of further investigation into therapeutic strategies aimed at disrupting condensate formation in Ewing sarcoma and other related malignancies.

## Graphical abstract

Ewing sarcoma is driven by the EWS::FLI1 fusion protein, which disrupts gene expression by hijacking the BAF complex via ARID1A. ARID1A's ability to form biomolecular condensates is crucial for tumor growth, making it a potential therapeutic target. However, targeting these transient condensates is challenging, requiring further research.

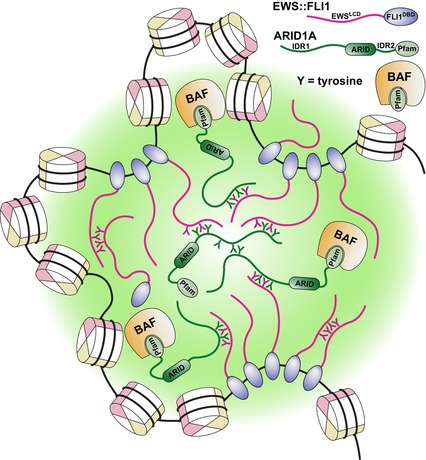

**Abbreviations**
ARID1A: AT‐rich interactive domain‐containing protein 1A
BAF complex: BRG1/BRM associated factor chromatin remodeling complex
EWS: RNA‐binding protein EWS
EWS::FLI1: fusion between EWS and Friend leukemia integration 1 transcription factor
IDRs: intrinsically disordered regions
MLOs: membraneless organelles
PrLDs: prion‐like domains

The cellular milieu is a crowded environment where myriad biochemical processes occur simultaneously. Our understanding of how this environment is spatially and temporally regulated is expanding, with membraneless organelles (MLOs) emerging as key components of intracellular organization [1]. MLOs are intracellular biomolecular condensates that form via liquid–liquid phase separation, driven by multiple weak, transient interactions between constituent molecules [1]. In proteins, these interactions are often mediated by intrinsically disordered regions (IDRs), which provide the multivalency necessary for multiple weak contacts that serve to accumulate intermolecular associations [2]. Prion‐like domains (PrLDs), a term originating from research on self‐associating yeast proteins [3], are commonly used to describe IDRs enriched in polar and aromatic amino acids that serve as key nucleators of MLOs [4, 5]. In healthy cells, the formation of MLOs is critical for various cellular processes, such as in transcription condensates, where RNA polymerase II associates with machinery like chromatin remodelers, transcription factors, and splicing factors to coordinate gene expression [1]. However, the same mechanisms driving these processes in healthy cells can be hijacked to promote disease pathogenesis [1].

In Ewing sarcoma, the oncogenic fusion protein EWS::FLI1 acts as a pseudo‐transcription factor, altering gene expression through condensate‐mediated interactions with the BRG1/BRM associated factor (BAF) chromatin remodeling complex [6, 7]. The prevailing view is that EWS::FLI1, via the PrLD of EWS, recruits the BAF complex to GGAA‐microsatellites and target gene enhancer sites, thereby opening chromatin, facilitating recruitment of transcriptional machinery, enhancing aberrant gene expression, and promoting oncogenesis [6, 7] (Fig. 1). Several BAF complex components contain PrLDs that form condensates both *in vitro* and intracellularly, and these PrLDs co‐localize to condensates containing the PrLD of FUS, a protein with a similar sequence composition to EWS and also involved in oncogenic fusions [5, 8]. The AT‐rich interactive domain‐containing protein 1A (ARID1A), the DNA‐binding core subunit of the BAF complex, has been identified as the key BAF complex component mediating the interaction with EWS::FLI1, specifically through its N‐terminal PrLD [9]. In a recent issue of *Nature Communications*, work by Kim et al. reinforced ARID1A's role as a key cofactor in EWS::FLI1‐driven oncogenesis, proposing that it acts as a central nucleator of aberrant condensates in Ewing sarcoma. Additionally, the study described a direct interaction between EWS::FLI1 and ARID1A that could represent a therapeutic vulnerability [10] (Fig. 1).

**Fig. 1:**
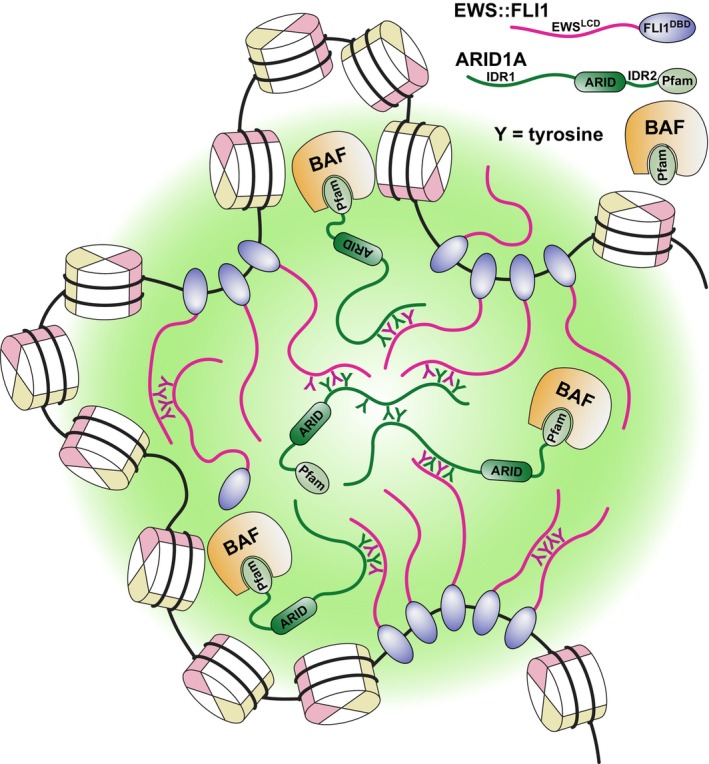
Tyrosine residues in the EWS and ARID1A intrinsically disordered regions (IDRs) interact, providing the main driving force for biomolecular condensate formation and localize recruitment and assembly of the BAF complex at EWS::FLI1‐mediated GGAA‐microsatellites and specific gene enhancer sites. These interactions also facilitate long‐range contacts that promote unique chromatin 3D structures. Tyrosine residues shown are illustrative and do not represent all the tyrosine residues found in either the ARID1A or EWS::FLI1 IDRs.

The BAF complex is well‐established as a key facilitator of aberrant gene expression in Ewing sarcoma, providing access to oncogenes targeted by EWS::FLI1 [6, 7]. Consequently, several BAF subunits, including ARID1A, are overexpressed in Ewing sarcoma [10]. The authors investigate the mechanistic basis for ARID1A's involvement, revealing that tumor growth is significantly influenced by ARID1A overexpression and is correlated with its ability to form intracellular condensates. This overexpression leads to an increased abundance of nuclear BAF‐containing condensates, with the resulting aberrant gene expression being dependent on ARID1A's condensate‐forming capability. The targeted genes, regulated by EWS::FLI1, promote tumor cell proliferation and migration, further supporting the established finding that EWS::FLI1 interfaces with the BAF complex in the context of a biomolecular condensate [7, 9].

Tyrosine‐tyrosine interactions have been previously identified as key drivers of PrLD condensate formation [4, 5]. The EWS PrLD relies on contacts between tyrosine residues to facilitate homotypic condensate formation, and these tyrosines are also crucial for EWS::FLI1's interaction with the BAF complex [4, 7]. In the case of ARID1A, tyrosine residues within its two PrLDs drive condensate formation, while its structured Pfam domain recruits other BAF subunits. Accordingly, EWS::FLI1 colocalizes with ARID1A condensates through direct interactions involving these tyrosines. These findings complement previous work showing that tyrosine residues within the EWS PrLD of EWS::FLI1 are essential for its oncogenic activity and interact with the BAF complex [7, 11]. Moreover, intracellular condensate formation by ARID1A enhances EWS::FLI1 condensate formation, demonstrating a cooperative mechanism between EWS::FLI1 and the BAF complex that promotes aberrant gene expression, mediated by ARID1A.

These findings by Kim et al. provide mechanistic insight into how EWS::FLI1 hijacks the BAF complex to drive oncogenesis, addressing a long‐standing question in the field. ARID1A's newly revealed role as the key interface between EWS::FLI1 and the BAF complex highlights the need for developing and investigating inhibitors targeting ARID1A [10]. With tumor growth significantly hindered by loss of condensate‐competent ARID1A, targeting ARID1A could prove effective as part of a combination therapy for Ewing sarcoma. Additionally, other members of the BAF complex are also overexpressed in Ewing sarcoma, but the effect of their functional loss has yet to be investigated. Beyond ARID1A's direct interaction with EWS::FLI1, other BAF subunits may perform Ewing‐specific functions making them promising therapeutic targets as well. Given that ARID1A is specific to the cBAF subtype and all three BAF complex subtypes are known to interact with EWS onco‐fusions, there is still much to learn about the relationship between EWS::FLI1 and BAF complexes [12].

The work by Kim et al. [10] further reinforces that condensate formation and MLOs are central features of Ewing sarcoma tumorigenesis. EWS::FLI1's ability to form aberrant condensates appears to be involved in nearly every disrupted process in Ewing sarcoma, and impairing condensate formation significantly reduces the oncogenic potential of EWS::FLI1 and its cofactors. However, inhibiting condensate formation in any disease remains a daunting challenge, as the transient interactions that enable condensate formation are not well suited for high‐affinity, specific targeting by small molecules. Much work remains to be done to understand how to effectively target diseases where condensate formation plays a crucial role, such as Ewing sarcoma and related malignancies.

## Conflict of interest

The authors declare no conflict of interest.

## Author contributions

EJS and DSL conceived, wrote, edited, and revised the manuscript.
